# Enhanced Cardiac Function in Gravin Mutant Mice Involves Alterations in the β-Adrenergic Receptor Signaling Cascade

**DOI:** 10.1371/journal.pone.0074784

**Published:** 2013-09-18

**Authors:** Ashley N. Guillory, Xing Yin, Cori S. Wijaya, Andrea C. Diaz Diaz, Abeer Rababa’h, Sonal Singh, Fatin Atrooz, Sakthivel Sadayappan, Bradley K. McConnell

**Affiliations:** 1 Department of Pharmacological and Pharmaceutical Sciences, University of Houston Texas Medical Center, Houston, Texas, United States of America; 2 Department of Cell and Molecular Physiology, Stritch School of Medicine, Loyola University Chicago, Maywood, Illinois, United States of America; Brigham & Women's Hospital - Harvard Medical School, United States of America

## Abstract

Gravin, an A-kinase anchoring protein, targets protein kinase A (PKA), protein kinase C (PKC), calcineurin and other signaling molecules to the beta2-adrenergic receptor (β_2_-AR). Gravin mediates desensitization/resensitization of the receptor by facilitating its phosphorylation by PKA and PKC. The role of gravin in β-AR mediated regulation of cardiac function is unclear. The purpose of this study was to determine the effect of acute β-AR stimulation on cardiac contractility in mice lacking functional gravin. Using echocardiographic analysis, we observed that contractility parameters such as left ventricular fractional shortening and ejection fraction were increased in gravin mutant (gravin-t/t) animals lacking functional protein compared to wild-type (WT) animals both at baseline and following acute isoproterenol (ISO) administration. In isolated gravin-t/t cardiomyocytes, we observed increased cell shortening fraction and decreased intracellular Ca^2+^ in response to 1 µmol/L ISO stimulation. These physiological responses occurred in the presence of decreased β_2_-AR phosphorylation in gravin-t/t hearts, where PKA-dependent β_2_-AR phosphorylation has been shown to lead to receptor desensitization. cAMP production, PKA activity and phosphorylation of phospholamban and troponin I was comparable in WT and gravin-t/t hearts both with and without ISO stimulation. However, cardiac myosin binding protein C (cMyBPC) phosphorylation site at position 273 was significantly increased in gravin-t/t versus WT hearts, in the absence of ISO. Additionally, the cardioprotective heat shock protein 20 (Hsp20) was significantly more phosphorylated in gravin-t/t versus WT hearts, in response to ISO. Our results suggest that disruption of gravin’s scaffold mediated signaling is able to increase baseline cardiac function as well as to augment contractility in response to acute β-AR stimulation by decreasing β_2_-AR phosphorylation and thus attenuating receptor desensitization and perhaps by altering PKA localization to increase the phosphorylation of cMyBPC and the nonclassical PKA substrate Hsp20.

## Introduction

Activation of the beta-adrenergic receptors (β-ARs) via the sympathetic nervous system is the primary mechanism through which cardiac contractility and output is modulated [1]. Briefly, norepinephrine or epinephrine binds to β-ARs to activate GTP-binding proteins, which regulate the activity of adenylyl cyclase. Stimulatory GTP-binding proteins (G_s_) prompt the formation of cAMP, which activates protein kinase A (PKA), the main effector of β-AR signaling. PKA phosphorylates an assortment of proteins involved in the regulation of calcium movement and contractility. Conversely, inhibitory G-proteins (G_i_) attenuate adenylyl cyclase activity resulting in the reduction of PKA-mediated regulation of cardiac function [2,3].

There are three types of β-ARs in the myocardium: β_1_-, β_2_-, and β_3_-ARs. Approximately 70-80% of the β-ARs in the heart is comprised of β_1_-ARs, which couple to G_s_; while β_2_-ARs, which make up 20-30% of the total β-ARs, can couple with both G_s_ and G_i_. Less than 10% of the total β-ARs are β_3_-ARs which couple to the G_i_/nitric oxide pathway to depress cardiac contractility [4]. Additionally, the three receptors also have distinct subcellular locations. For example, β_1_-ARs have been shown to be evenly distributed throughout the plasma membrane while β_2_-ARs tend to be localized primarily in the caveolae. Thus, differential effects of the β-AR subtypes can have profound effects on the activation and activity of PKA.

PKA is a heterotetramer consisting of two catalytic subunits and two regulatory subunits. Binding of cAMP to the regulatory subunits causes the activation and release of the catalytic subunits [5]. The catalytic subunits phosphorylate proteins involved in a variety of cellular processes including gene expression and contractility [6]. PKA phosphorylates many proteins involved in excitation-contraction coupling such as cardiac troponin I (cTnI), ryanodine receptor, cardiac myosin binding protein C (cMyBPC) and the L-type calcium channel [2,7-9]. PKA’s ability to phosphorylate a wide variety of proteins to fine tune contractility is associated with its binding to A-kinase anchoring proteins (AKAPs). AKAPs bind to the PKA regulatory subunit dimer and target PKA to specific subcellular locations [10]. Over 70 AKAPs have been identified and 14 of these are found in the myocardium. Each AKAP localizes PKA with a specific subset of substrates that also bind to the scaffolding protein. For example, AKAP 15/18 binds with L-type calcium channels while mAKAP binds phosphodiesterase 4D3, calcineurin and the ryanodine receptor [11,12].

Gravin, also known as AKAP12, SSeCKS or AKAP250, is an AKAP that is highly expressed in the heart. In addition to scaffolding PKA, gravin binds protein kinase C (PKC), calcineurin and other signaling molecules along with the β_2_-AR [13,14]. Gravin has been shown to be a key factor in the desensitization/resensitization cycle of the receptor as both PKA and PKC can phosphorylate β_2_-AR, which leads to its desensitization [15]. Suppression of gravin expression or disruption of the gravin/PKA interaction has been shown to alter the cycling of the receptor [16,17]. Additionally, it has been shown that there was a reduction in the recruitment of proteins involved in receptor desensitization such as G-protein-coupled receptor kinase 2 (GRK2) and β-arrestin to the β_2_-AR in the absence of gravin [16,17]. Although the role of gravin in β_2_-AR desensitization has been extensively investigated *in vitro*, the effect of gravin’s absence of functional protein on cardiac function in the presence of β-AR stimulation *in vivo* has not been investigated. Furthermore, we have previously reported that general inhibition of AKAP/PKA interactions results in increased cardiac function following acute β-AR stimulation [18]. Therefore, we hypothesized that similar results would be observed in the absence of functional gravin upon β-AR stimulation and, because of gravin’s relationship with β_2_-AR, that cardiac function would also be increased in the absence or presence of β-AR stimulation. Thus, to test the effect of mice lacking functional gravin protein (designated gravin-t/t; *where t refers to truncated alleles*) – that does not express the critical region required for β_2_-AR, PKA or PKC binding – we studied cardiac function as well as the β-AR signaling pathway in gravin-t/t mice after infusion with the nonspecific β-AR agonist isoproterenol.

## Methods

### Generation of Mutant Gravin (gravin-t/t) Mice.

**Figure 1:**
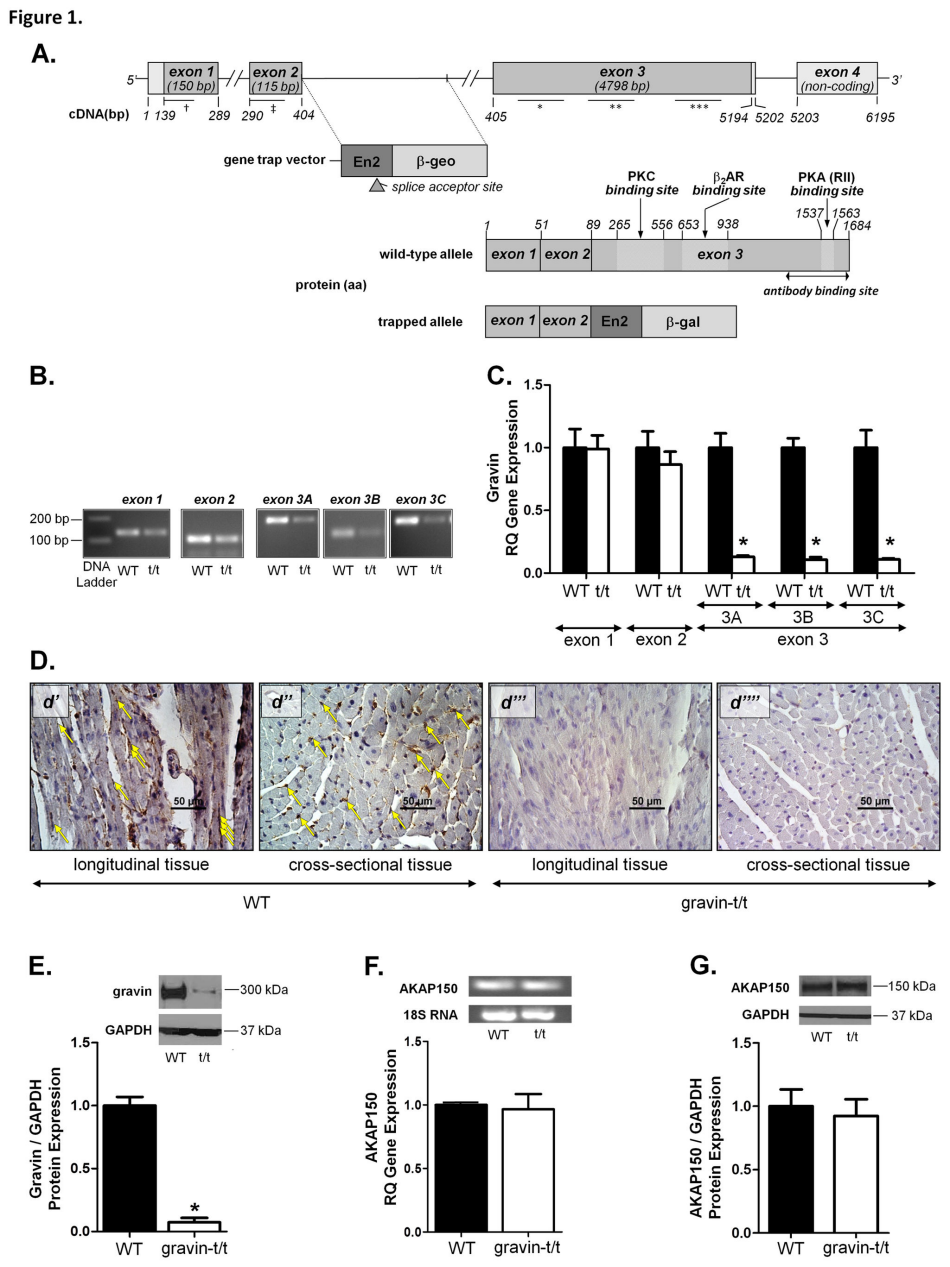
Disruption of the Akap12 (gravin) gene and the expression of the gravin in WT and gravin-t/t mice. (*A*) The strategy to disrupt the *Akap12* (gravin) gene (NM_031185) using gene trap technology. The ES cell line (XE450), containing genetically engineered retroviral gene trap, was obtained from BayGenomics. The gene trap vector integrated into the chromosome within the *Akap12* gene (gene trap vector, En2:β-gal), immediately following exon-2. *Akap12* gene sequence includes the relative primer set binding site, represented by a bar for the PCR construct (†: exon 1; ‡: exon 2; *: exon 3A; **: exon 3b; ***: exon 3c). Gravin protein sequence includes the relative binding site of the gravin antibody (AKAP250/gravin antibody; Santa Cruz), represented by an arrowed bar marking the antibody’s epitope (*Gene and protein sequence diagrams are not drawn to scale*). Gravin gene and protein expression levels were determined in hearts of WT and gravin-t/t mice. (*B*) Amplified PCR products and (*C*) RT-qPCR analysis of the exons of the gravin gene. (*D*) Immunohistochemistry (IHC) analysis of gravin protein expression in heart tissue sections from WT and gravin-t/t mice. Heart sections show positively stained cardiomyocytes that expressed gravin from WT (*d*’ longitudinal tissue section; *d*’’ cross-sectional tissue) mice (*yellow arrows*), but not from gravin-t/t (*d*’’“longitudinal tissue section; *d*”’*’’* cross-sectional tissue) mice. This positively gravin-stained expression in WT tissue sections is identified as dark brown oval clusters near the sarcolemma (*yellow arrows*). (*E*) Western blot analysis of gravin protein expression levels in LV homogenates from WT and gravin-t/t mice (*n*=4-6). (*F*) RT-qPCR analysis of total heart RNA identified the AKAP150 gene using AKAP150 specific primers in WT and gravin-t/t mouse hearts (*n*=4-7) and (*G*) Western blot analysis of AKAP150 protein expression levels in LV homogenates from WT and gravin-t/t mice (*n*=4-6). Experiments were performed in triplicate. The bar graphs show gene expression data collected from 4 to 7 mice per group and Western blot data collected from 4 to 6 mice per group. *Yellow* *arrows* in panels *d*’ and *d*’’ represent a sample of the ubiquitously expressed gravin in WT heart sections. Data are expressed as the mean ± S.E.M.; *, p<0.05 versus respective WT (panels c-d); t/t = gravin-t/t mice.

Gravin mutant mice were produced using gene trap technology to ablate the *Akap12* (gravin) gene (NM_031185). The embryonic stem (ES) cells containing the gravin trapped vector were obtained from BayGenomics. As shown in Figure 1A, the *Akap12* gene, which contains 4 exons, was engineered to delete exons 3 and 4 by the insertion of a specific gravin gene trap vector into intron 2. This gravin gene trap vector contained the splice-acceptor sequence (within the En2 domain) upstream of the reporter genes, which is a fusion of β-galactosidase (β-gal) and neomycin phosphotransferase II (β-geo). The resulting insertional mutation created a fusion transcript containing sequences from exons upstream to the insertion, joined to the β-geo marker, thus disrupting the *Akap12* gene. This cell line (XE450; BayGenomics) had been successfully expanded and re-sequenced to confirm the ES cell line identity, microinjected into normal blastocysts and surgically implanted into foster mice, which subsequently gave birth to mice with a chimeric (coat color) phenotype at the University of Maryland School of Medicine Transgenic / Knockout Core Facility. The offspring of germline- transmitting chimeric mice are of ES (XE450) origin. Seven-week-old male chimeric mice were mated with female wild-type mice (C57BL/6) to produce litters containing wild-type and heterozygous mice. From this mating, heterozygous mice were crossed (*Het x Het* mating) to produce homozygous mutant and wild-type mice. β-gal was used as the reporter gene, identifying this fusion product together with the splice acceptor/En2 exon sequence of the gene trap. The gene trap was previously determined to be located within intron 2 of the *Akap12* gene (gravin), *data not shown*. Our results demonstrate germline transmission of the targeted gene trap to be within the mouse *Akap12* gene, as shown by the successful PCR amplification of a 681bp fragment of the β-gal reporter gene, using mouse tail genomic DNA obtained from the *Het x Het* mating (*data not shown*). This 681bp product was obtained using the β-gal gene specific forward (5’-TTA TCG ATG AGC TGG TGG TTA TGC-3’) and reverse (5’-GCG CGT ACA TCG GGC AAA TAA TAT C-3’) primers. Homozygous gravin mutant mice (designated gravin-t/t; *where t refers to truncated alleles*) resulting from germline transmission of the gravin targeting gene trap and wild-type mice were used to study the effects of targeted disruption of PKA localization to the β_2_-AR and ensuing cardiac function. Wild-type (WT) mice and homozygous mutant gravin mice are bred on the C57bl/6 background.

### Reverse Transcription Quantitative PCR (RT-qPCR)

Extraction of total RNA was performed from male mouse heart tissues using an RNeasy mini kit (QIAGEN) according to the manufacturer’s instructions and RNA were qualified by a spectrophotometer. Recombinant gravin cDNA was synthesized using random hexamers via the Invitrogen Superscript III Kit (Invitrogen) while the rest of the cDNA was synthesized using First Strand DNA Synthesis (Origene). Amplifications were performed using SYBR Green. PCR core reagents (Applied Biosystems) and transcript levels were quantified by using an ABI 7900 Sequence Detection System (Applied Biosystems). The mean Ct (cycle threshold) value of triplicate reactions was normalized against the mean ct value of GAPDH or 18s RNA. Primers were used at 2.5 µM. Gravin *forward primer* (5’-TGG GCA TCC TTC AAA AAG ATG-3’); gravin *reverse primer* (5’-ACC TTA AGC TCT TCT TCC TTG T-3’); exon *1 forward primer (5‘-ATG GGT GCA GGC AGT TCC-3'*)*; exon 1 reverse primer (5'-CGG GAT CTC CAG CTG CTC-3'*)*; exon 2 forward primer (5’-CTC CCA CAG AAG AAT GGT CAG-3'*)*; exon 2 reverse primer (5'-GAC TTC TTC CTC TTG CCC ATC-3'*)*; exon 3A forward primer (5‘-GAG CAG GAG ACC ACC AAG AG-3'*); exon *3A reverse primer* (*5'-TTC TCC ATC TTT GGC TGC TT-3'*); exon *3B forward primer* (*5’-TGG GCA TCC TTC AAA AAG ATG-3’*) exon *3B reverse primer* (*5’-CCT TAA GCT CTT CTT CCT TGT-3’*); exon *3C forward primer* (*5’-GCC AGT GAA GAA CAT GAG CA-3'*); exon *3C reverse primer* (*5'-TGC AAT CTG CTT TGT CTT GG-3'*); AKAP150 *forward primer* (5’- AGG ATG GGG CTC TTC CTA AG-3'); AKAP150 *reverse primer* (5'-GGG TCT GGG CTT TTA TCT CC-3'); GAPDH *forward primer* (5’-GTC TCC TCT GAC TTC AAC AGC G-3’); GAPDH *reverse primer* (5’-ACC ACC CTG TTG CTG TAG CCA A-3’); 18s RNA *forward primer* (5’-TCA AGA ACG AAA GTC GGA GG-3’); and 18s RNA *reverse primer* (5’-GGA CAT CTA AGG GCA TCA C-3’). The PCR products from gravin-t/t and WT mice were loaded onto 0.8% agarose gels and then visualized.

### Echocardiography and ECG Measurements

Baseline measurements by echocardiography and ECG were obtained before intraperitoneal (i.p.) injection with isoproterenol (ISO; 0.25 µg/g), a β-AR agonist, or ascorbic acid (vehicle; 0.002%). Cardiac morphology and function were assessed by serial M-mode echocardiography with a VisualSonics Vevo 770 High-Resolution In-Vivo Micro-Imaging System (VisualSonics In, Ontario, Canada) equipped with a 30 MHz microprobe. Ventricular measurements in M-mode were taken at baseline and 3 minutes after ISO injection with at least three readings per mouse.

### Isolation of Adult Mouse Ventricular Myocytes, Contractility Assays and Ca^2+^ Measurements

Adult mouse ventricular myocytes were isolated from the hearts of adult male gravin-t/t and WT mice (16 to 20 weeks old). Cardiac contractility and intracellular Ca^2+^ were measured by an IonOptix system as previously described [19]. Briefly, isolated cardiomyocytes were incubated with 1µM Fura-2 acetoxymethyl ester (Fura 2-AM, Invitrogen) in loading buffer for 15min at room temperature, and then washed 3 times with loading buffer. After 3 washes, the cells were placed in a Plexiglas chamber and perfused continuously with cardiomyocyte loading buffer at 37°C. The cells were paced at 1Hz with IonOptix MyoPacer Field stimulator (pulse duration 1ms; 5 volts). The ratio of the intracellular Ca^2+^ kinetics was measured in the absence or presence of ISO (1µmol/L) for 5min. Background fluorescence was subtracted from actual cardiomyocyte fluorescence by subtracting the data recording in the absence of cells from the data recording in the presence of a cell, while keeping all other parameters constant at the end of each cell recording measurement. Data were analyzed using IonWizard (IonOptix) software using low-pass butterworth filter at frequency of 60. Measurements included baseline, peak (total peak – baseline), time to 50% of peak, time from peak to 50% decay, time to 90% of peak and time from peak to 90% decay.

### cAMP production

cAMP production was measured according to manufacturer’s instructions using an enzyme immunoassay kit (Enzo LifeSciences) in membrane fractions as previously described [20]. Each experiment was performed in duplicate. cAMP production was normalized to milligrams of protein.

### PDE activity

PDE activity was determined via an enzyme immunoassay kit (Enzo LifeSciences) using cytosolic preparations according to the manufacturer’s instructions. Samples were desalted using column chromatography. Each experiment was performed in triplicate. Data were normalized to milligrams of protein used.

### PKA Activity

PKA activity was determined via an enzyme immunoassay kit (Enzo LifeSciences) using cytosolic preparations according to the manufacturer’s instructions. Each experiment was performed in triplicate. Data were normalized to milligrams of protein used. Specific PKA activity was determined using 10µM peptide inhibitor of PKA (PKI).

### Western Blot Analysis

Western blot was carried out as previously described [18]. Immunoblot analysis was carried out as previously described using antibodies for phospho-Hsp20 (Abcam), total- and phospho-phospholamban, total- and phospho-troponin I, GAPDH (Cell Signaling), total Hsp20 (Millipore), PDE4D (Pierce antibodies), AKAP250/gravin, AKAP150, β_1_-AR, β_2_-AR, phospho-β_2_-AR (Santa Cruz), phospho-MyBPC (Ser-273), phospho-MyBPC (Ser-282), phospho-MyBPC (Ser-302) and total-MyBPC (provided by co-author). Crude heart homogenates were resolved by SDS-PAGE gradient (4–12% Bis-Tris) gels, and then transferred to polyvinylidine difluoride membranes. Blots were then incubated overnight at 4°C with primary antibodies. The blots were washed with TBS containing 0.1% Tween 20 (TBST), and then probed with the appropriate HRP-conjugated secondary antibodies (anti-mouse; anti-rabbit; Cell Signaling). The signal was detected by using SuperSignal West Pico Chemiluminescent Substrate (Thermo Scientific). Densitometric analyses of the immunoblots were performed by ImageJ Data Acquisition Software, National Institute of Health, Bethesda, MD. For each substrate, the anti-phospho-protein antibody signal was normalized to the total-protein antibody signal. The graphs show the ratio of phosphorylated to total protein normalized to WT vehicle.

### Immununohistochemistry Analysis

Hearts were collected from euthanized animals for immunohistochemical studies. Animals were first heparinized, to prevent blood clotting prior to being anesthetized with 3% isoflurane, for 10 minutes. The chest cavity was opened and the heart was excised, connective tissue removed, rinsed in PBS and blotted. The heart tissue was then fixed with 10% formalin and paraffin embedded. Sections were cut at 8 µm thickness and stained using Vector Labs products according to the manufacturer’s instructions. The AKAP250 / gravin antibody (Santa Cruz) was used to identify the gravin protein. The brown color was developed using DAB (3, 3’-diaminobenzidine) peroxidase substrate kit and hematoxylin was used as a counterstain, identifying the cell nuclei. The heart sections were imaged with an inverted microscope (Nikon Eclipse Ti-U) and digital camera (Nikon Digital Sight DS-Qi1Mc).

### Histological Analysis

Hearts were harvested and fixed in 10% formalin overnight, embedded in paraffin, sectioned at 5 µm thickness and stained using hematoxylin and eosin (H&E) and Masson’s Trichrome (MT) according to manufacturer’s instructions. The sectional area was measured with a microscope (Nikon Eclipse Ti-U) and digital camera (Nikon Digital Sight DS-Qi1Mc). Photomicrographs of the sections were evaluated for morphology and cellular dimensions.

### Statistical Analysis

Data were processed using Microsoft Excel and GraphPad Prism 5.0. All values are expressed as the mean ± S.E.M. Comparisons between two groups were determined using unpaired 2-tailed Student’s *t* test. Analysis was performed using one-way ANOVA, followed by a Tukey’s *post hoc* multiple comparison test when multiple groups were compared. *P* values of less than 0.05 were considered significant.

### Ethics Statement - Study Approvals and Consent

All animal studies have been approved by the Institutional Animal Care and Use Committee (IACUC) and ethics committee at the University of Houston (UH; # UH-ACP-11-032) and the Baylor College of Medicine (BCM; # BCM-AN-5199). Animal care was provided for in AAALAC accredited animal barrier facilities at UH and BCM, located within the Texas Medical Center (TMC) and have therefore been performed in accordance with the ethical standards laid down in the 1964 Declaration of Helsinki and its later amendments. Also, all authors of this report gave their informed consent prior to their inclusion in the study.

## Results

### Expression of Gravin in WT and Mutant Gravin-t/t Mice

The *Akap12* (gravin) gene (NM_031185; *mouse*) was disrupted using gene trap technology. Gene trapping technology is a high-throughput method of generating knockout mouse clones, utilizing technology that uses genetically engineered retroviruses that infect mouse ES cells *in vitro*, integrating into the chromosome of the cell and disrupting the function of the specific gene into which it integrates. Specifically, the ES cell line (XE450; BayGenomics) that contained the genetically engineered retroviral gene trap, had integrated into the chromosome within the *Akap12* gene (Figure 1A; *gene trap vector*). Thus, this method permitted the generation of mice lacking functional gravin protein.

The mutant gravin allele in these mice contains the gene trap vector that contains a splice acceptor site immediately following exon 2, which causes early termination of the transcript. This mutant allele removes the remaining two 3’ exons (exon 3 and exon 4). Gravin gene expression in gravin-t/t mice was characterized and confirmed by reverse transcription quantitative PCR (RT-qPCR). The gravin transcript was quantified using SYBR Green and gravin specific forward and reverse primers for exons 1, 2 and 3; amplified from total RNA isolated from the LV of WT and gravin-t/t mice. Figure 1C shows that exon 3, which encodes approximately 95% of the *Akap12* gene, was significantly reduced (^≈^90%), when quantified at three different regions within the exon. Exons 1 and 2 of the mutant allele are expressed at wild-type levels. As a control to show equivalent gene expression and cDNA amplification using a standard housekeeping gene, GAPDH was quantified and confirmed to be the same between WT and gravin-t/t mice. Amplified PCR products from each of these regions are shown in Figure 1B.

To determine the amount of gravin protein expressed in WT mice and gravin-t/t mice, the gravin protein was quantified by Western blot analysis and immunohistochemistry. Anti-gravin antibody specific to the carboxyl-terminal amino acids (Figure 1A) identified a protein in WT and gravin-t/t LV protein homogenates that was significantly reduced in gravin-t/t LV protein homogenates (Figure 1E); whereas the monoclonal anti-GAPDH antibody confirmed equal endogenous levels of the 37-kDa housekeeping GAPDH protein in WT and gravin-t/t mice. Protein studies have demonstrated that this mutant allele produces less than 10% of the normal amount of gravin (Figure 1E; *n*=4-6). Also, the mutant allele is predicted to encode from the amino-terminal to amino acid residue 89 of the 1684 residues from wild-type gravin, representing less than 6% of the full length protein. Additionally, to further characterize gravin protein expression in WT and gravin-t/t mice, we performed immunohistochemistry analysis on heart tissue sections. As illustrated in Figure 1D, heart sections show positively stained cardiomyocytes expressing gravin from WT mice, but not in gravin-t/t mice. These results demonstrate that the ubiquitously expressed gravin that is associated with cell membranes of WT hearts is absent in gravin-t/t hearts. Differences in the detection of residual gravin protein expression in gravin-t/t hearts by Western blot versus immunohistochemistry analysis may be due to differences in sample preparation and/or the antibody’s epitope / gravin protein recognition due to the difference in epitope presentation between experimental applications. These results are in agreement with a recently published study utilizing a gravin gene trap mouse model to investigate gravin’s role in synaptic plasticity and memory. The authors also reported a 10% or less gravin protein expression in their mouse model which they attributed to another isoform of gravin that is not targeted to the plasma membrane (also known as gravin-β) [21].

AKAP150 is a smaller molecular weight protein that shares many of gravin’s binding partners such as the β_2_-AR, PKC and PP2B [22]. AKAP150 is also expressed in the heart; thus, we measured AKAP150 gene expression by RT-qPCR (Figure 1F; *n*=4-7) and AKAP150 protein expression by Western blot (Figure 1G; *n*=4-6) to confirm that AKAP150 expression remained unchanged in the gravin-t/t mice. Therefore, in gravin-t/t mice, the differences in cardiac scaffold mediated function and signaling is not due to differences in AKAP150 expression. In this study, homozygous gravin-t/t mice do not significantly express the critical region required for β_2_-AR, PKA or PKC binding that are encoded by exon 3 and thus do not express functional gravin protein (Figure 1A-D). Therefore, this gravin-t/t mouse model allowed for us to uniquely test the effect of targeted disruption of PKA localization to the β_2_-AR and ensuing cardiac function *in vivo* in response to acute β-AR stimulation.

### Increased Cardiac Function in Mutant Gravin-t/t Mice

**Figure 2:**
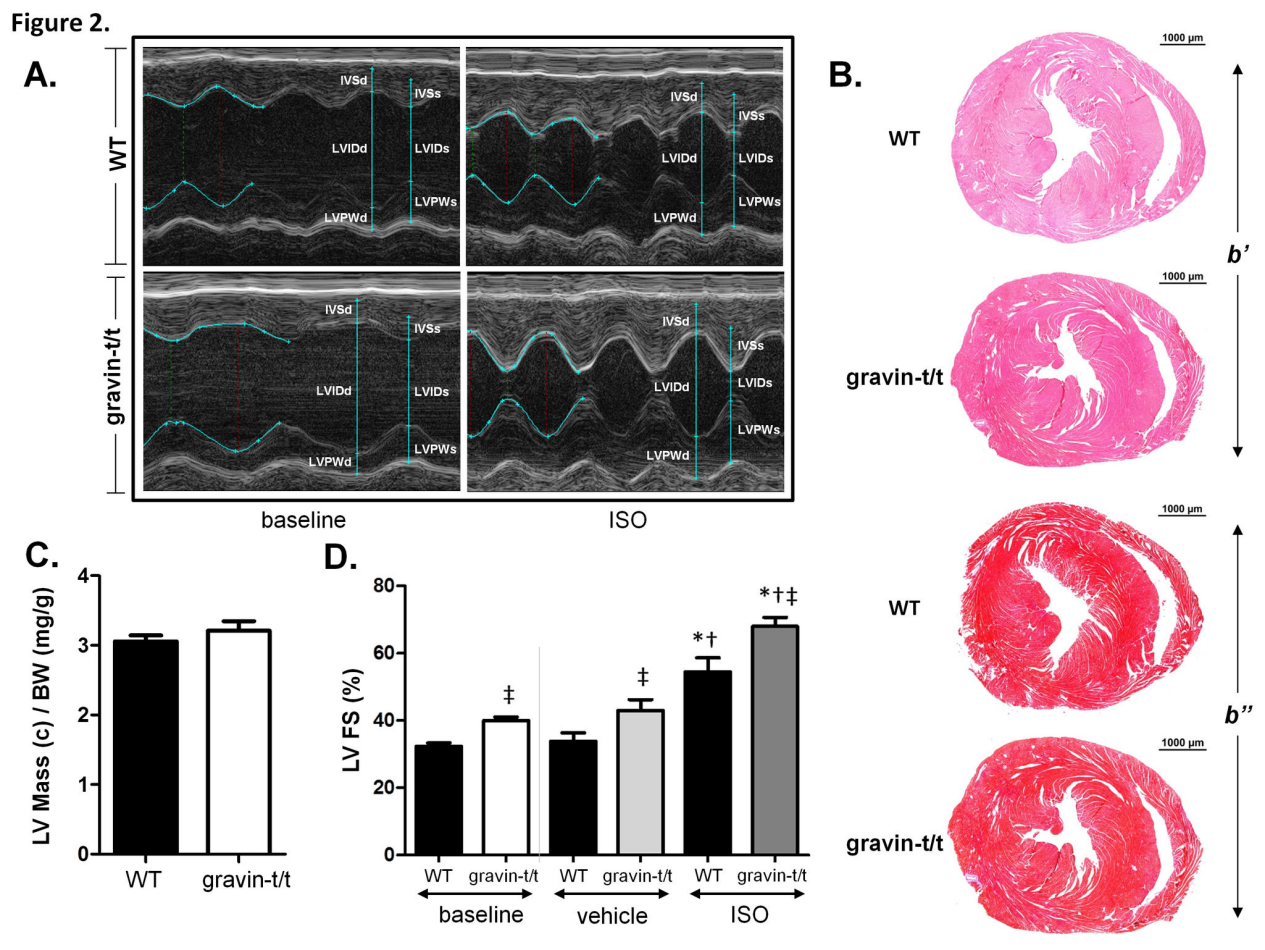
Cardiac morphology in response to acute ISO stimulation in WT and gravin-t/t mice. (*A*) Representative transthoracic echocardiographic M-mode image of the LV from WT and gravin-t/t hearts at baseline and following 3 minutes of ISO infusion; measured echocardiographic parameters and the M-mode wall tracings for the first few cycles, are shown in each panel in *turquoise* (*B*) Hematoxylin and eosin (H&E; panel *b*’) and Masson’s trichrome (MT; panel *b*″) stained paraffin sections of WT and gravin-t/t hearts. (*C*) LV mass (corrected) to body weight (BW) ratio (mg/g) illustrating cardiac size of WT and gravin-t/t hearts 3 minutes of ISO infusion. (*D*) Percentage of left ventricular fractional shortening (LVFS) as derived from transthoracic echocardiographic M-mode images of the ventricle in WT and gravin-t/t hearts at baseline and following 3 minutes of ISO infusion. Vehicle mice were infused with control buffer. Data are expressed as the mean ± S.E.M.; baseline measurements, n=36 (WT) and n=36 (gravin-t/t); all other measurements, n=9; *p<0.05 vs. baseline of same genotype; † p<0.05 either baseline in WT vs. baseline in gravin-t/t mice or ISO in WT vs. ISO in gravin-t/t mice; ‡p<0.05 either baseline in WT vs. ISO in gravin-t/t mice or ISO in WT vs. baseline in gravin-t/t mice.

To examine the effect of mice lacking functional gravin protein on β-adrenergic signaling, WT and gravin-t/t mice were injected i.p. with isoproterenol (0.25µg/g). Echocardiography was used to assess cardiac dimensions and function in WT and gravin-t/t mice at baseline and three minutes after ISO injection (Figure 2). Left ventricular mass (corrected) for both WT and gravin-t/t hearts was determined via echocardiography and normalized to body weight (mg/g). There was no significant difference in left ventricular mass to body weight ratio between the WT and the gravin-t/t mice (Figure 2C). Additionally, histological analysis did not reveal any differences between WT and gravin-t/t hearts (Figure 2B).

The cardiac dimensions intra-ventricular septum (IVS) and left ventricular (LV) posterior wall (PW) were also measured at baseline and 3 minutes after injection during both systole and diastole (Figure 2A and Table 1). IVS and LVPW were not significantly different at diastole between the two groups. However, both IVS and LVPW at diastole were significantly increased in both WT and gravin-t/t mice following ISO injection compared to the vehicle treated animals. Conversely, IVS and LVPW at systole were significantly increased in the gravin-t/t mice even at baseline despite the absence of stimulation. Left ventricular internal dimensions (LVID) were also measured (Table 1). At diastole, no difference was observed between WT and gravin-t/t mice. However, at systole, LVID was significantly decreased in the gravin-t/t mice. Collectively, these data indicate that there is increased tension at contraction in the gravin-t/t mice and would be expected to result in improved cardiac function. With ISO stimulation, both IVS and LVPW thickness were increased in WT and gravin-t/t hearts compared to their respective controls. Additionally, both parameters were significantly increased in the gravin-t/t ISO hearts compared to the WT ISO hearts, indicating that the absence of functional gravin improves contractile function (Table 1). Although, LVIDs was significantly decreased in both WT and gravin-t/t mice following ISO infusion, there was not a significant difference between WT and gravin-t/t ISO treated mice. A representative M-mode echocardiography depicting these changes in cardiac dimensions is shown in Figure 2A.

**Table 1:** Echocardiographic Parameters of WT and Gravin-t/t Mice.

	**WT Baseline** (*n*=36)	**Gravin-t/t Baseline** (*n*=36)	**WT Vehicle** (*n*=9)	**Gravin-t/t Vehicle** (*n*=9)	**WT ISO** (*n*=9)	**Gravin-t/t ISO** (*n*=9)
**HR (bpm**)	478 ± 9	467 ± 6	467 ± 20	448 ± 20	596 ± 8*	571 ± 16*
**IVSd (mm**)	0.88 ± 0.02	0.87 ± 0.02	0.92 ± 0.03	0.93 ± 0.03	1.13 ± 0.15*‡	1.05 ± 0.22*‡
**IVSs (mm**)	0.96 ± 0.02	1.13 ± 0.02†	1.08 ± 0.05	1.30 ± 0.07†	1.12 ± 0.03*	1.43 ± 0.08*†
**LVIDd (mm**)	4.20 ± 0.06	4.23 ± 0.06	4.11 ± 0.23	3.98 ± 0.10	3.47 ± 0.13*	3.74 ± 0.12
**LVIDs (mm**)	2.78 ± 0.07	2.40 ± 0.07†	2.67 ± 0.26	2.55 ± 0.16	1.66 ± 0.22*‡	1.33 ± 0.14*‡
**LVPWd (mm**)	0.81 ± 0.03	0.83 ± 0.04	0.78 ± 0.09	0.78 ± 0.07	1.10 ± 0.04*	1.09 ± 0.04*
**LVPWs (mm**)	1.18 ± 0.03	1.44 ± 0.04†	1.08 ± 0.09	1.30 ± 0.11†	1.84 ± 0.12*	2.05 ± 0.10*†
**SV (µL**)	40.63 ± 0.79	50.96 ± 0.49†	43.94 ± 4.01	51.30 ± 2.36†	47.62 ± 1.16‡	64.25 ± 4.06*†‡
**EF (%**)	58.86 ± 1.25	68.89 ± 1.25†	65.40 ± 4.15	75.42 ± 3.21†	87.81 ± 3.38*	93.94 ± 1.28*†‡

All values are expressed as mean ± SEM; ISO, isoproterenol; HR, heart rate; IVS, intraventricular septum; LVID, left ventricular internal dimensions; LVPW, left ventricular posterior wall; SV, stroke volume; EF, ejection fraction; s, systole; d, diastole; *p<0.05 vs. baseline of same genotype; † p<0.05 either baseline in gravin-t/t vs. baseline in WT mice, or vehicle in gravin-t/t vs. vehicle in WT mice, or ISO in gravin-t/t vs. ISO in WT mice; ‡p<0.05 either ISO in WT vs. baseline in gravin-t/t mice, or ISO in gravin-t/t vs. baseline in WT mice. Statistical analysis was performed using a one-way ANOVA with a Tukey’s post hoc test.

Cardiac contractility was also assessed by echocardiography at both baseline and after i.p. injection in WT and gravin-t/t mice. Heart rate was similar between WT and gravin-t/t mice at baseline and both similarly increased with ISO treatment (WT baseline: 478.0±8.6; gravin-t/t baseline: 467.0±6.4; WT ISO: 596.4±8.1; gravin-t/t ISO: 570.7±16.4; bpm; Table 1). The cardiac contractility parameters LV ejection fraction (EF), LV fractional shortening (FS) and stroke volume (SV) were significantly increased in gravin-t/t mice even in the absence of ISO stimulation (Figure 2D and Table 1). After ISO stimulation, these parameters increased in both treatment groups, versus their respective controls. LV fractional shortening was also significantly enhanced in the gravin-t/t ISO mice compared to WT ISO mice (WT baseline: 32.40±0.92; gravin-t/t baseline: 40.02±1.04; WT ISO: 54.47±4.16; gravin-t/t ISO: 68.03±2.67; %; p<0.0001; Figure 2D). Similarly, stroke volume was much greater in the gravin-t/t ISO mice than the WT ISO mice (WT baseline: 40.63±3.79; gravin-t/t baseline: 50.96±0.49; WT ISO: 47.62±1.16; gravin-t/t ISO: 64.25±4.06; µl; p=0.0056; Table 1). These data indicate that hearts lacking functional gravin protein have increased contractility both before and after β-AR stimulation.

### Decreased Cardiomyocyte Intracellular Ca^2+^ Transients and Increased Cardiomyocyte Contractility in Response to ISO stimulation

**Figure 3:**
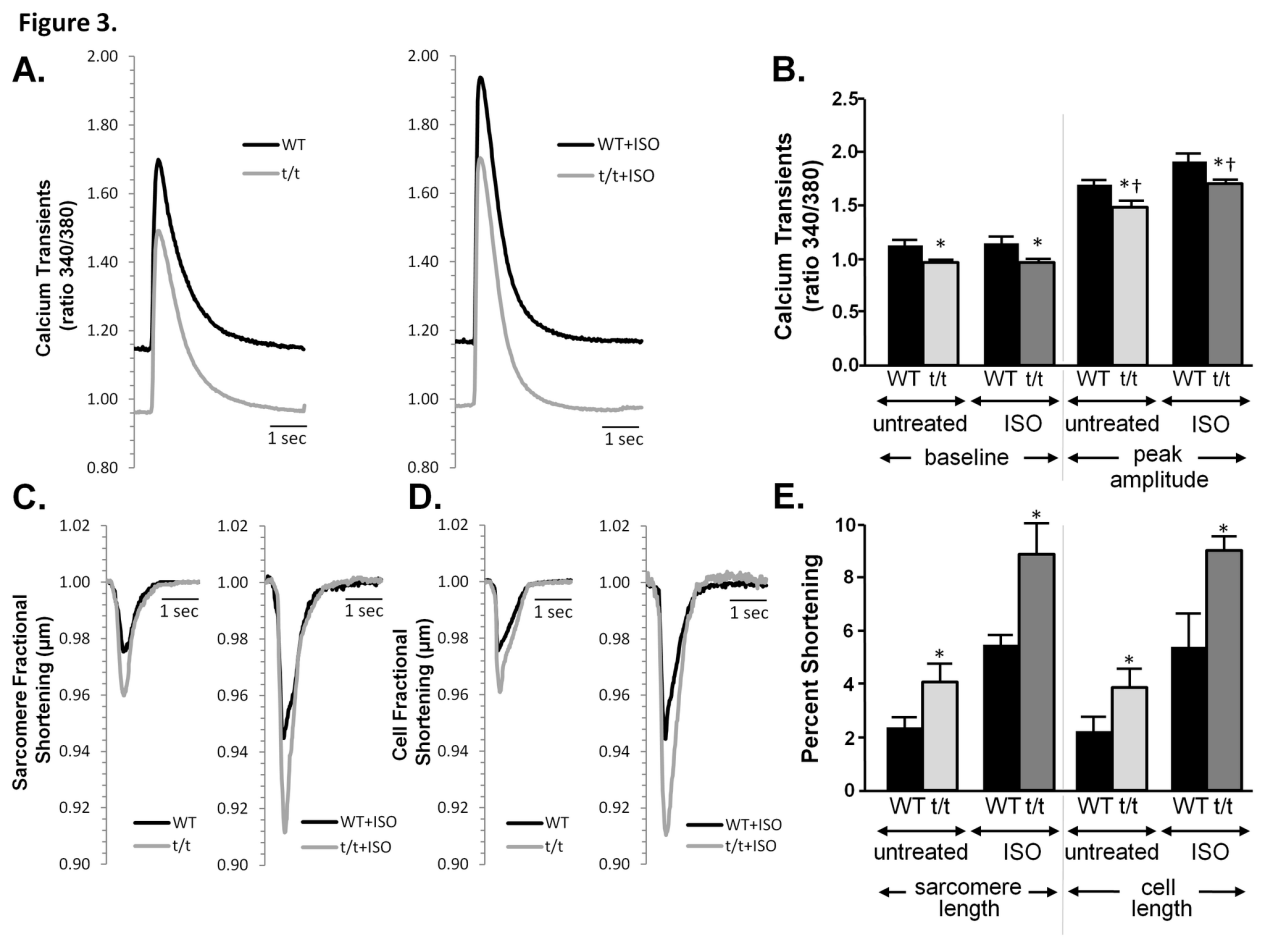
Calcium transients, sarcomere length shortening and cell length shortening in response to ISO stimulation in WT and gravin-t/t cardiomyocytes. (*A*) Representative cardiomyocyte Ca^2+^ transients and (*B*) corresponding bar graph of the 340/380 ratio in WT (n=18) and gravin-t/t (n=30) myocytes perfused with control buffer and WT (n=14) and gravin-t/t (n=29) myocytes perfused with ISO. (*C*) Representative cardiomyocyte sarcomere length fractional shortening transients of WT (n=9) and gravin-t/t (n=13) myocytes perfused with control buffer and WT (n=8) and gravin-t/t (n=8) myocytes perfused with ISO. (*D*) Representative cell length fractional shortening transients of WT (n=12) and gravin-t/t (n=12) myocytes perfused with control buffer and WT (n=13) and gravin-t/t (n=10) myocytes perfused with ISO. (*E*) Shows the corresponding bar graph of the percent shortening in sarcomere length and cell length of the represented traces in *Panel*’s *C* and *D*. The bar graphs show the Ca^2+^ transient 340/380 ratio (Panel *B*) and percent shortening (Panel *E*) from cardiomyocytes collected from 4 to 6 mice per group. Data are expressed as the mean ± S.E.M.; *, p<0.05 versus respective WT (Panel *B*); *, p<0.01 versus respective WT (Panel *E*); t/t = gravin-t/t mice; †, p<0.05 either baseline in WT vs. baseline in gravin-t/t mice or ISO in WT vs. ISO in gravin-t/t mice.

Since gravin-t/t mice had significantly enhanced cardiac function as measured by echocardiography, we next investigated whether mutant gravin-t/t altered cardiomyocyte contractility and Ca^2+^ cycling. Studies have shown that contractile dysfunction is often associated with alterations in intracellular Ca^2+^ homeostasis [23]. Therefore, we hypothesized that the absence of functional gravin positively impacted contractile function by more efficient cycling of intracellular Ca^2+^ transients. To test this hypothesis, isolated cardiomyocytes from WT and gravin-t/t mice were loaded with Fura-2 to image cytosolic Ca^2+^ transients and where the sarcomere and cell length fractional shortening were concurrently measured (Figure 3). As shown in Figure 3A-B, untreated gravin-t/t cardiomyocytes displayed a significant decrease in baseline Ca^2+^ and peak amplitude of the intracellular Ca^2+^ transient, as compared to WT cardiomyocytes. In response to 1 µmol/L (µM) ISO stimulation, a proportionally similar decrease in baseline Ca^2+^ and peak amplitude of the intracellular Ca^2+^ transient was also observed in gravin-t/t cardiomyocytes. These observations were further supported by the baseline and peak (total peak – baseline) measurements of WT and gravin-t/t cardiomyocytes shown in Table 2. Similar to the *in vivo* echocardiographic cardiac function observations (Figure 2D and Table 1), both untreated and ISO stimulated gravin-t/t cardiomyocytes showed significantly increased contractility, as determined by percent sarcomere fractional shortening and percent unloaded cell length fractional shortening compared to the respective WT cardiomyocytes (Figure 3C-E). Additionally, Ca^2+^ transient measurements of WT and gravin-t/t cardiomyocytes were further characterized, illustrating the time to 50% of peak, time to 90% of peak, time from peak to 50% decay and time from peak to 90% decay (Table 2). No differences were observed in the Ca^2+^ transient time to peak or the Ca^2+^ transient time from peak to decay (or baseline) between WT and gravin-t/t cardiomyocytes (Table 2). Correspondingly, no differences were observed in the sarcomere or cell fractional shortening time to peak (either 50% or 90% of peak) or time from peak to baseline (either 50% or 90% decay) between WT and gravin-t/t cardiomyocytes (*data not shown*). Thus, cardiomyocytes isolated from gravin-t/t mice have enhanced cardiomyocyte contractility in the presence of proportionally lower diastolic baseline and maximum height of intracellular Ca^2+^ transients. However, the rate of rise and the rate of decline of the Ca^2+^ transient, sarcomere fractional shortening or cell fractional shortening were not significantly different between WT and gravin-t/t cardiomyocytes, either in the absence or presence of ISO stimulation. These data indicate that disruption of the scaffolding protein gravin increases cardiomyocyte function, and thus cardiac function, perhaps by altering PKA signaling to modulate Ca^2+^ homeostasis.

**Table 2:** Calcium Measurements of WT and Gravin-t/t Cardiomyocytes.

	**WT** (*n*=18)	**Gravin-t/t** (*n*=30)	**WT ISO** (*n*=14)	**Gravin-t/t ISO** (*n*=29)
**Baseline (340/380**)	1.128 ± 0.043	0.939 ± 0.025*	1.060 ± 0.039	0.870 ± 0.019*
**Peak (340/380**)	1.589 ± 0.082	1.438 ± 0.035*	1.870 ± 0.046†	1.686 ± 0.042*†
**Time to 50% peak (seconds**)	0.016 ± 0.001	0.015 ± 0.001	0.016 ± 0.001	0.013 ± 0.001
**Time to 90% peak (seconds**)	0.027 ± 0.002	0.0297 ± 0.002	0.030 ± 0.002	0.022 ± 0.001
**Time to 50% baseline (seconds**)	0.166 ± 0.014	0.152 ± 0.008	0.154 ± 0.005	0.135 ± 0.004
**Time to 90% baseline (seconds**)	0.405 ± 0.036	0.367 ± 0.031	0.351 ± 0.023	0.298 ± 0.028

All values are expressed as mean ± SEM; ISO, isoproterenol; *n*=14-30 per group; † p<0.05 vs. baseline of same genotype; *p<0.05 either baseline in WT vs. baseline in gravin-t/t mice or ISO in WT vs. ISO in gravin-t/t mice

### β-AR Expression

**Figure 4:**
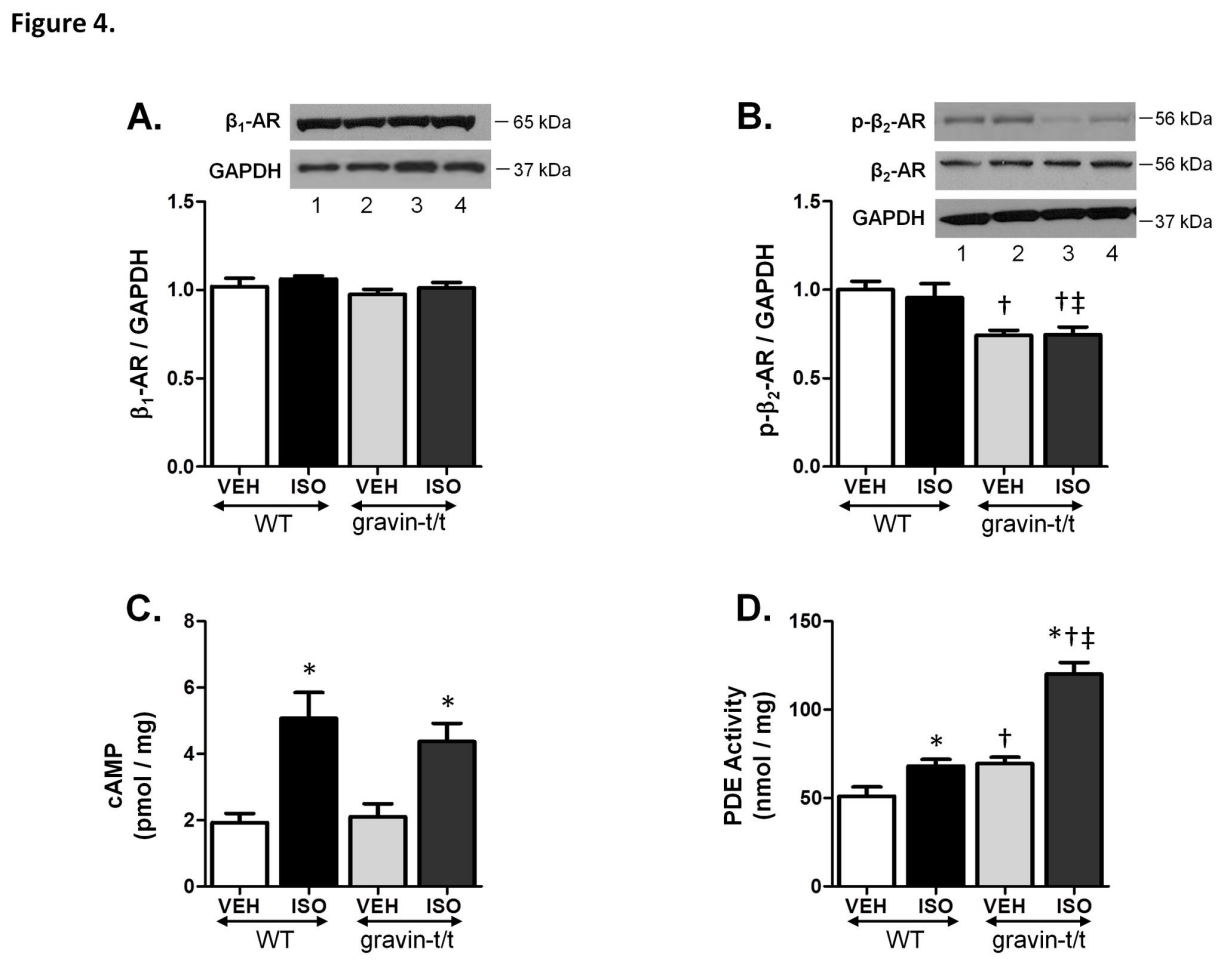
β-adrenergic receptor expression, cAMP production and PDE activity in WT and gravin-t/t left ventricles. Western blot analysis of (*A*) β_1_-AR protein expression levels and (*B*) β_2_-AR protein expression levels in left ventricular membrane fractions isolated from WT and gravin-t/t mice following acute vehicle or ISO infusion (10µg/g/min). For each substrate, the upper panel shows a Western blot with anti-protein antibody and the lower panel shows a Western blot with an antibody to GAPDH (Lane 1: WT VEH; Lane 2: WT ISO; Lane 3: gravin-t/t VEH; Lane 4: gravin-t/t ISO). The bar graphs show the ratio of the receptor expression to GAPDH normalized to WT vehicle. (*C*) cAMP production in left ventricular membrane fractions isolated from WT and gravin-t/t mice following acute vehicle or ISO infusion (10µg/g/min). (*D*) PDE activity in left ventricular cytosolic fractions isolated from WT and gravin-t/t mice following acute vehicle or ISO infusion (10µg/g/min). Data are expressed as the mean ± S.E.M.; n= 6-8; *P <0.05 vs. baseline of same genotype; † p<0.05 either baseline in WT vs. baseline in gravin-t/t mice or ISO in WT vs. ISO in gravin-t/t mice; ‡p<0.05 either baseline in WT vs. ISO in gravin-t/t mice or ISO in WT vs. baseline in gravin-t/t mice.

C.C. Malbon’s group has definitively shown the role of gravin in β_2_-AR’s desensitization and resensitization cycle and that suppression of gravin expression results in decreased desensitization of the receptor *in vitro* [13,16]. We examined via Western blot whether absence of functional gravin altered the protein expression levels of both β_1_-AR and β_2_-AR. Our results indicate that β_1_-AR protein expression levels were similar between WT and gravin-t/t animals and that ISO stimulation does not alter the receptor’s expression levels (Figure 4A). Similarly, β_2_-AR protein expression levels were similar between WT and gravin-t/t animals (Figure 4B). Since gravin has been shown to be a key factor in the desensitization / resensitization cycle of the β_2_-AR, and that PKA can phosphorylate the receptor which leads to its desensitization, we therefore measured β_2_-AR phosphorylation in WT and gravin-t/t heart homogenates. Our results show that β_2_-AR phosphorylation is significantly decreased in gravin-t/t versus WT hearts, both in the absence and presence of acute ISO stimulation (Figure 4B). These data indicate that the enhanced contractility in the gravin-t/t mice is not due to altered β-AR protein expression levels, but rather due to decreased β_2_-AR phosphorylation.

### cAMP Production and PDE Activity and Expression

β-ARs stimulate adenylyl cyclase to enhance the production of cAMP, which activates signaling cascades through PKA and other signaling molecules such as Epac1 [24]. Since mice lacking functional gravin protein are expected to inhibit β_2_-AR signaling, we expected that cAMP production would be altered in the gravin-t/t mice. However, our results indicate that cAMP production between WT and gravin-t/t untreated mice are similar. ISO stimulation, as expected, increased cAMP production in both WT and gravin-t/t mice but again there was no difference between the two groups (Figure 4C).

**Figure 5:**
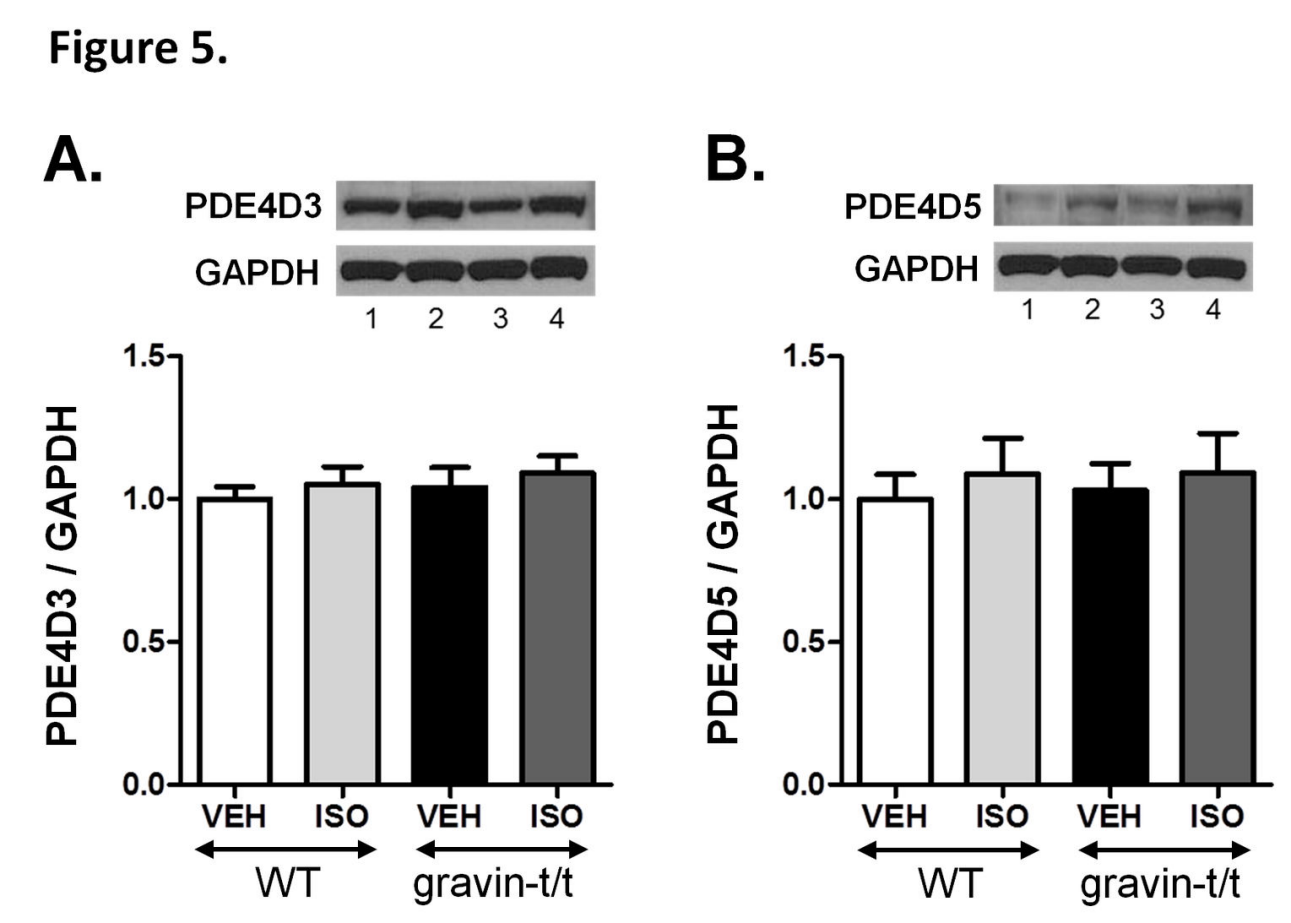
PDE4D3 and PDE4D5 protein expression levels in WT and gravin-t/t mice. (*A*) Western blot analysis of PDE4D5 in heart homogenates isolated from WT and gravin-t/t mice following acute vehicle or ISO infusion (10µg/g/min; Lane 1: WT VEH; Lane 2: WT ISO; Lane 3: gravin-t/t VEH; Lane 4: gravin-t/t ISO). The *upper* *panel* shows a Western blot with anti-PDE4D5 antibody and the *lower* *panel* shows a Western blot with an antibody to GAPDH. (*B*) Western blot analysis of PDE4D3 in heart homogenates isolated from WT and gravin-t/t mice following acute vehicle or ISO infusion (10µg/g/min); (Lane 1: WT VEH; Lane 2: WT ISO; Lane 3: gravin-t/t VEH; Lane 4: gravin-t/t ISO). The *upper* *panel* shows a Western blot with anti-PDE4D3 antibody and the *lower* *panel* shows a Western blot with an antibody to GAPDH. The *bar* *graphs* show the ratio of PDE4D isoform to GAPDH normalized to WT vehicle. Data are expressed as the mean ± S.E.M.; n= 6-8 samples.

Phosphodiesterases (PDE) act to terminate PKA dependent signaling by degrading cAMP [25]. Additionally, PDE4D activity has been shown to significantly impact cardiac function as PDE4D knockout mice have increased contractility [26]. Gravin has been shown to include PDE4D isoforms 3 and 5 in its scaffolding complex [27]. Therefore, we measured total PDE activity as well as PDE4D3 and PDE4D5 protein expression levels. As expected, PDE activity significantly increased in WT mice following ISO exposure. However, baseline PDE activity in the gravin-t/t mice was significantly higher than that of WT control mice. In fact, PDE activity in the gravin-t/t vehicle control mice equaled that of WT ISO treated mice. Furthermore, gravin-t/t mice treated with ISO had significantly higher PDE activity than both the controls and the WT ISO group (p<0.0001; Figure 4D). Both PDE4D5 and PDE4D3 protein expression levels were similar between WT and gravin-t/t mice regardless of treatment (Figure 5A-B). This data suggests that the increase in PDE activity is not due to increased expression levels of PDE4D3 and PDE4D5, the PDE isoforms purported to bind to gravin [27]. However, it is possible that PDE activity, similar to that of PKC, is inhibited upon binding to gravin [28]. Thus, the removal of gravin could enhance PDE activity without altering PDE protein expression levels. Alternatively, enhanced β-AR function could increase the cAMP/PKA induced activation of PDE, which would allow for enhanced contractility without altering the total cAMP level. In essence, the increased cAMP production expected with enhanced β-AR activity would be balanced by a corresponding increase in PDE activity.

### ISO-stimulated Increases in PKA Activity and Substrate Phosphorylation

**Figure 6:**
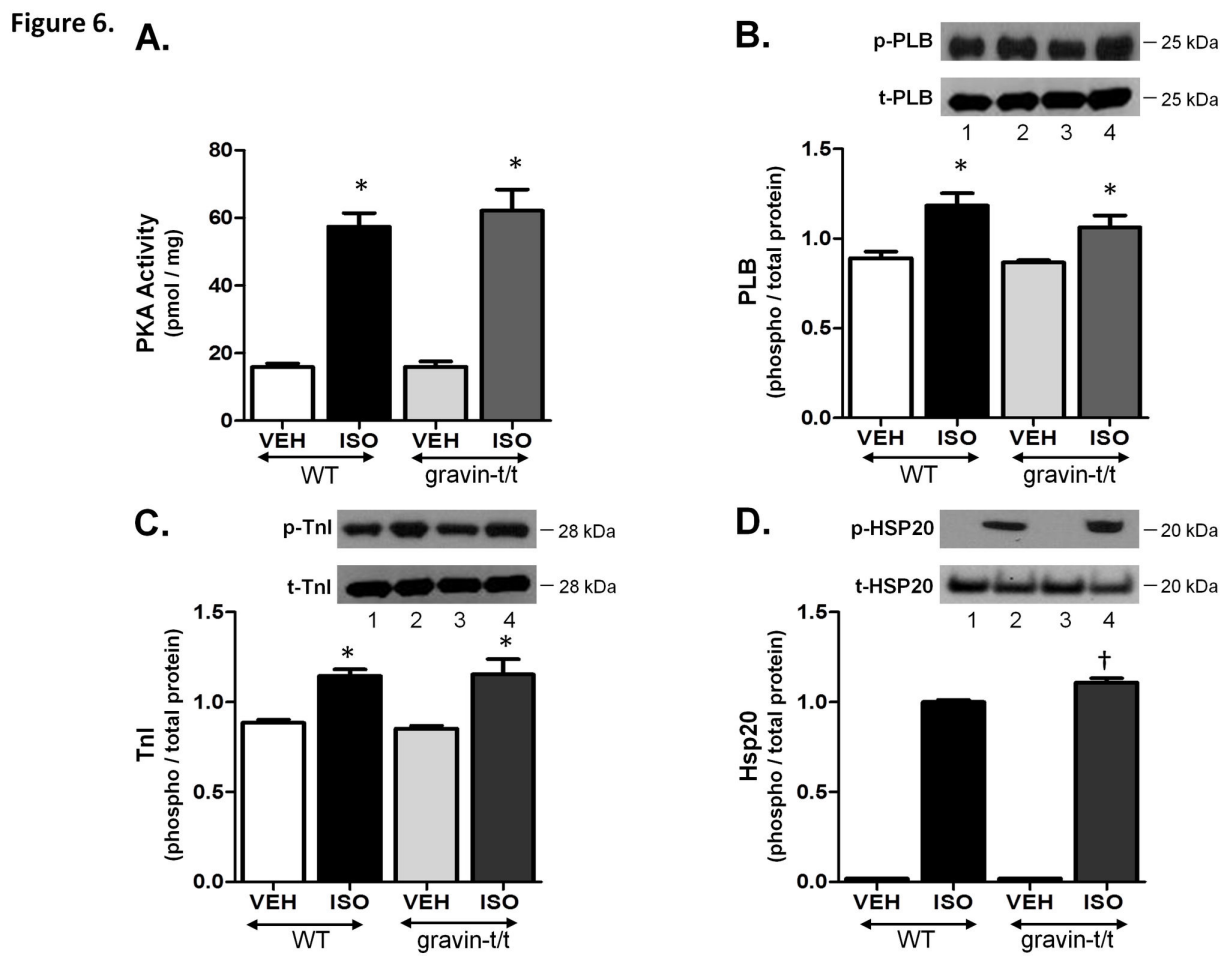
PKA activity and substrate phosphorylation in response to acute ISO stimulation in WT and gravin-t/t mice. (*A*) PKA activity in left ventricular cytosolic fractions isolated from WT and gravin-t/t mice following acute vehicle or ISO infusion (10ug/g/min). Western blot analysis of PKA substrate phosphorylation of (*B*) phospholamban (PLB), (*C*) cardiac troponin I (TnI) and (*D*) heat shock protein 20 (Hsp20) in heart homogenates isolated from WT and gravin-t/t mice following acute vehicle or ISO infusion (10µg/g/min). For each substrate, the upper panel shows a Western blot with anti-phospho-protein antibody and the lower panel shows a Western blot with an antibody to total-protein (Lane 1: WT VEH; Lane 2: WT ISO; Lane 3: gravin-t/t VEH; Lane 4: gravin-t/t ISO). The bar graphs show the ratio of phosphorylated to total protein normalized to WT vehicle. Data are expressed as the mean ± S.E.M.; n= 4 to 6 samples; *p<0.05 vs. baseline of same genotype; † p<0.05 either baseline in WT vs. baseline in gravin-t/t mice or ISO in WT vs. ISO in gravin-t/t mice; ‡p<0.05 either baseline in WT vs. ISO in gravin-t/t mice or ISO in WT vs. baseline in gravin-t/t mice.

Since PKA is a major effector of β-AR stimulation and the phosphorylation of its substrates has profound effects on the regulation of contraction and relaxation [2], we evaluated PKA activity in vehicle and ISO treated WT and gravin-t/t mice hearts. Additionally, we expected that PKA activity would be reduced in the gravin-t/t mice hearts due to the increase of PDE activity. However, similar to what was seen in the cAMP production a comparable increase in PKA activity was seen in both WT and gravin-t/t ISO stimulated hearts indicating the absence of β_2_-AR and PKA binding to gravin does not affect the activation of the kinase despite enhanced PDE activity (Figure 6A). Since PKA activity was not altered in the gravin-t/t mice, we hypothesized that perhaps PKA localization is altered and that this alteration would affect PKA substrate phosphorylation. Thus, the level of phosphorylation of phospholamban (PLB) and cardiac troponin I (cTnI) was measured (Figure 6B-C).

PKA-induced PLB phosphorylation was significantly increased in both WT and gravin-t/t hearts following ISO infusion (Figure 6B). When phosphorylated at Ser-16, PLB can no longer inhibit the sarcoplasmic reticulum Ca^2+^ ATPase, which is responsible for the uptake of Ca^2+^ into the sarcoplasmic reticulum following contraction. Reuptake of Ca^2+^ into the sarcoplasmic reticulum results in faster cardiac relaxation as well as increases the Ca^2+^ store available for release at the next depolarization of the cell. Similarly, cTnI phosphorylation was also increased in the ISO infused hearts of both genotypes (Figure 6C). cTnI phosphorylation is also associated with augmenting relaxation of the heart as increased phosphorylation of this protein reduces the sensitivity of the myofilaments to Ca^2+^. Since PKA phosphorylation of these two proteins was similar in the WT and gravin-t/t mice, these data indicate that gravin does not play an essential role in mediating their phosphorylation by PKA.

Since we did not see changes between WT ISO and gravin-t/t ISO in PLB and cTnI, the classical substrates of PKA, we investigated whether the absence of gravin’s functional binding domain affected other PKA substrates. Phosphorylation of heat shock protein 20 (Hsp20) by PKA has been shown to be cardioprotective in several pathophysiological disease states including cardiac hypertrophy and cardiac ischemia/reperfusion injury. Additionally, phosphorylation of this protein significantly increases as cAMP levels rise as a result of β-AR stimulation [29]. Hsp20 is normally complexed with PDE4D, which reduces its phosphorylation [30,31]. Gravin has also been shown to complex PDE4D, therefore we hypothesized that the absence of gravin’s functional binding domain would alter PDE4D localization and thus enhance Hsp20 phosphorylation [27]. Although, phosphorylated Hsp20 was not detectable in either the WT or gravin-t/t vehicle hearts, Hsp20 phosphorylation was significantly increased in the gravin-t/t ISO group compared to the WT ISO (Figure 6D).

**Figure 7:**
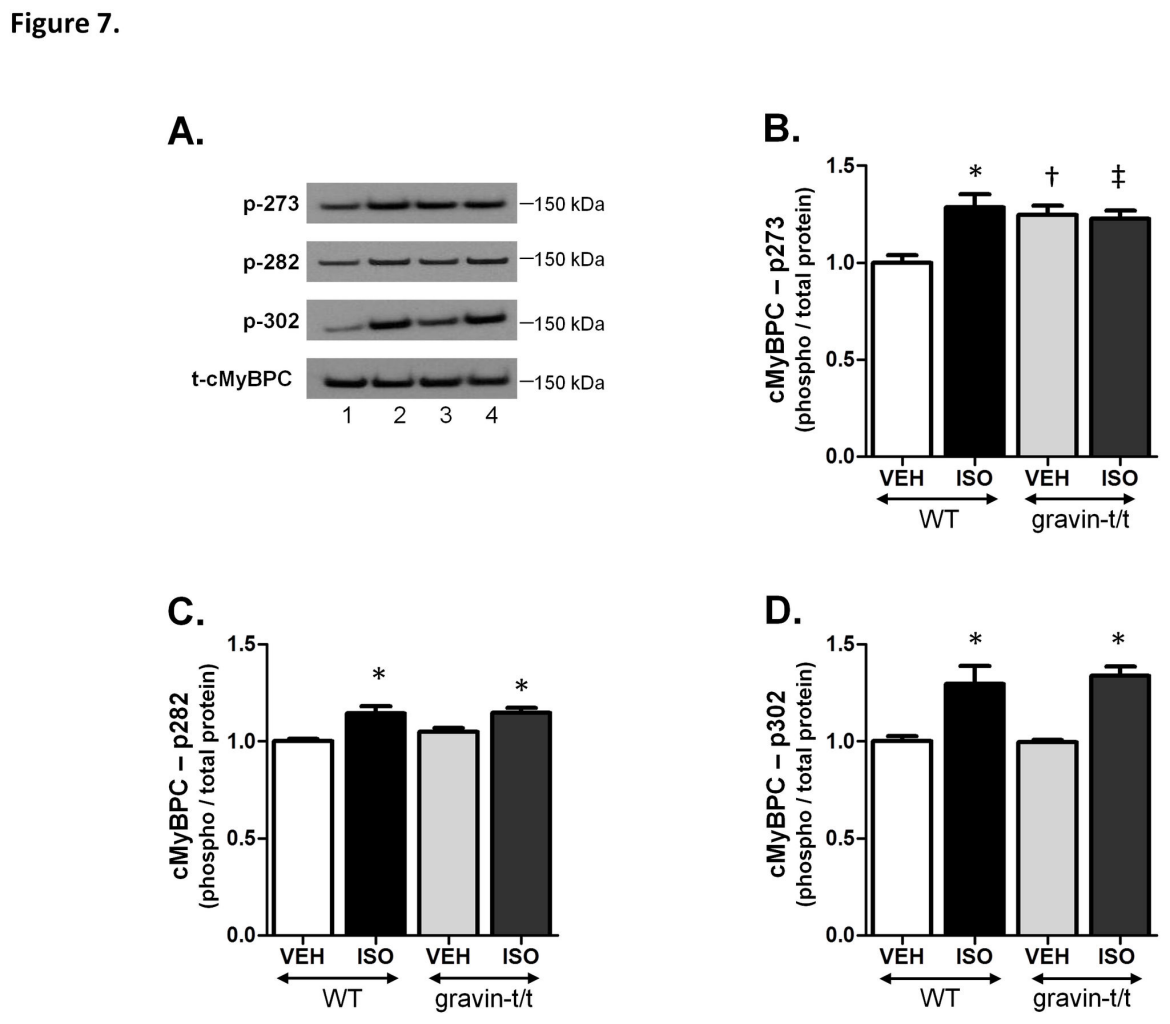
Phosphorylation of cardiac Myosin Binding Protein C in response to acute ISO stimulation in WT and gravin-t/t mice. (*A*) Representative western blots of the three phosphorylation sites of cardiac myosin binding protein C (cMyBPC) as well as total cMyBPC in heart homogenates isolated from WT and gravin-t/t mice following acute vehicle or ISO infusion (10µg/g/min); (Lane 1: WT VEH; Lane 2: WT ISO; Lane 3: gravin-t/t VEH; Lane 4: gravin-t/t ISO). (*B*-*D*) The bar graphs show the ratio of phosphorylated to total protein for p273, p282 and p302 respectively normalized to WT vehicle. Data are expressed as the mean ± S.E.M.; n= 4 to 6 samples; *p<0.05 vs. baseline of same genotype; † p<0.05 either baseline in WT vs. baseline in gravin-t/t mice or ISO in WT vs. ISO in gravin-t/t mice; ‡p<0.05 either baseline in WT vs. ISO in gravin-t/t mice or ISO in WT vs. baseline in gravin-t/t mice.

We also measured the phosphorylation of cardiac myosin binding protein C (cMyBPC), which has three major phosphorylation sites, Ser-273, Ser-282 and Ser-302. These sites can be phosphorylated by a variety of proteins including PKA, PKC, CaMKII and protein kinase D [32-34]. cMyBPC is involved in the modulation of myofilament sensitivity to Ca^2+^ and ablation of these three phosphorylation sites has been shown to have decrease cardiac function [35-38]. Similar to what was seen with PLB and cTnI, phosphorylation of sites Ser-282 and Ser-302 in cMyBPC was significantly increased in both WT and gravin-t/t hearts in the presence of ISO stimulation compared to their respective controls with no difference between the two ISO groups (Figure 7). However, phosphorylation of site Ser-273 was significantly increased in the gravin-t/t vehicle treated hearts compared to the WT vehicle treated hearts but was not further augmented with ISO treatment. Following ISO stimulation, Ser-273 phosphorylation in WT hearts was increased to a level similar to that seen in the gravin-t/t animals (Figure 7A, B). These data indicate that cMyBPC phosphorylation at Ser-273 may play a key role in enhancing cardiac function in gravin-t/t mice. Our results correlate with recently published studies investigating the role of cMyBPC’s phosphorylation in cardiac function that demonstrated the importance of Ser-273 phosphorylation [39,40].

## Discussion

Cardiac contractility is largely dependent upon the movement of Ca^2+^ in which homeostasis is itself regulated mainly through β-AR signaling [1]. Although cardiac contractility is mainly mediated through augmentation of β_1_-AR signaling, signaling through β_2_-ARs coupled to G_s_ also has a positive inotropic effect. Additionally, signaling through β_2_-AR has been shown to be beneficial both by increasing contractility in the normal heart and also promoting cell survival and cardioprotection in the injured or failing heart [41]. Gravin facilitates the phosphorylation of the receptor by both PKA and PKC as well as the recruitment of GRK2 and β-arrestin to the β_2_-AR to initiate the desensitization/resensitization cycle and terminate the signaling of the receptor. Thus, blockade of the termination of β_2_-AR signaling would be expected to increase cardiac function. Furthermore, disruption of PKA/AKAP interactions by addition of Ht31, a peptide that competes with AKAPs to bind the PKA regulatory subunits, has previously been shown to have a positive inotropic effect in the presence of β-AR stimulation [18]. Accordingly, in our present study, using gravin-t/t mice, we demonstrate that removal of this anchoring protein’s domain required for β_2_-AR, PKA, and other signaling molecules has increased cardiac function following acute stimulation by the nonspecific β-agonist isoproterenol.

Our results demonstrate that cardiac contractility is significantly increased in gravin-t/t mice both before and after ISO stimulation. Since gravin is a key factor in the desensitization / resensitization cycle of the β_2_-AR, and this process is mediated by receptor phosphorylation, we hypothesized that decreased gravin mediated PKA-dependent β_2_-AR phosphorylation would result in decreased receptor desensitization. This decreased β_2_-AR desensitization would increase receptor availability and thus increase responsiveness to agonist (ISO) mediated enhanced cardiac contractility. In our experiments, we observed significantly lower β_2_-AR phosphorylation in gravin-t/t hearts. Therefore, our results suggest that disruption of gravin’s scaffold mediated signaling is able to increase baseline cardiac function as well as to augment contractility in response to acute β-AR stimulation by decreasing β_2_-AR phosphorylation and thus attenuating receptor desensitization.

Additionally, we had previously reported that global disruption of PKA/AKAP interactions, using adenoviral gene transfer of Ht31 in rat heart *in vivo*, resulted in a significant decrease of PKA phosphorylation of key proteins involved in excitation-contraction coupling, namely PLB, cTnI and the ryanodine receptor, after ISO stimulation [18]. Furthermore, Fink et al. has shown that adenoviral gene transfer of Ht31 into rat cardiomyocytes *in vitro* disrupted endogenous AKAP anchoring of PKA, resulting in decreased cTnI phosphorylation and *increased* contractile function to β-AR stimulation, which occurred in the *absence* of increased Ca^2+^ transients [42]. Similarly, in this report, we also now demonstrate that gravin-t/t cardiomyocytes resulted in increased contractile function, which occurred in the *absence* of increased Ca^2+^ transients indicating that the absence of gravin may act to increase Ca^2+^ sensitivity. In agreement with Fink et al., we suggest that the lack of change in the Ca^2+^ transients may involve altered Ca^2+^ influx through the L-type Ca^2+^ channel and/or altered Ca^2+^ release from SR through the ryanodine receptor as PKA phosphorylation modulates the activity of these two channels [42]. We also observed significantly lower baseline Ca^2+^ levels in the gravin-t/t mice. However, we did not see changes in PKA activity or PKA phosphorylation of PLB or cTnI that would be expected to alter Ca^2+^ homeostasis. Perhaps Ca^2+^ influx through the L-type Ca^2+^ channel or release from the SR through RyR is affected by altered PKA anchoring in the absence of functional gravin.

Surprisingly, despite enhanced contractility we did not observe the expected increases in cAMP production, PKA activity or PKA substrate phosphorylation in the gravin-t/t mice. However, new technology using AKAR, a fluorescent biosensor, to investigate the spatial and temporal dynamics of PKA activation and signaling have shown that preferential stimulation of β_2_-ARs results in differential phosphorylation of PKA substrates and appears to depend upon the proximity of the substrates to the kinase and the receptor [43]. This concept corroborates our current data as we did not see changes in phosphorylation of PLB, a PKA substrate that is distant from the receptor, but we did see enhanced phosphorylation of Hsp20, a protein that has been shown to be localized primarily in the cytosol much closer to the receptor, in the gravin-t/t ISO treated mice [30]. Previous studies have shown that Hsp20 phosphorylation is beneficial to the myocardium, therefore, this enhanced Hsp20 phosphorylation may contribute to the mechanism for increased cardiac function in the gravin-t/t mice [29]. Additionally, the lack of changes in PKA activity and PLB and cTnI phosphorylation in the gravin-t/t mice are comparable to recently published results concerning the role of gravin in synaptic plasticity and memory [21]. Havekes et al., found that the absence of PKA anchoring by gravin affected only a specific selection of PKA’s substrates and that these changes in phosphorylation were not accompanied by alterations in global PKA activity. However, these changes were sufficient to dampen PKA dependent forms of synaptic plasticity and memory [21].

Further investigation into PKA substrates revealed alterations in protein phosphorylation that may also play a role in mediating the increased cardiac function seen in the gravin-t/t mice. cMyBPC is an important contractile protein whose phosphorylation has recently been implicated in the control of cardiac function. cMyBPC is phosphorylated by a variety of proteins at three major sites: Ser-273, Ser-282 and Ser-302. Although it is known that these phosphorylation sites are essential for both the activity of the protein as well as normal cardiac function, the effect of altered phosphorylation at individual sites is not clearly understood [35,36,38]. However, recent studies have indicated that phosphorylation of Ser-273 is the key site required for modulating cardiac function [39,40]. Specifically, Jin et al. found that nitric oxide synthase (NOS1) enhances cardiac relaxation via myofilament calcium desensitization in hypertensive rat hearts and that upregulation of NOS1 increases cMyBPC Ser-273, in addition to cTnI (23/24) [40].

We have also observed that cMyPBC Ser-273 phosphorylation was significantly increased in the gravin-t/t mice compared to WT mice in the absence of ISO. We propose that this baseline increase in Ser-273 phosphorylation in the gravin-t/t mice may help to explain the increased cardiac function seen in the gravin-t/t mice as studies have shown that increased cMyBPC phosphorylation results in enhancement of the actin-myosin interaction resulting in the augmentation of the cross-bridge cycling rate and increased cardiac contractility [38,44,45]. However, our cMyBPC phosphorylation studies were limited to just three of the many phosphorylation sites present in this protein. A recently published study determined that there were at least seventeen human cMyBPC phosphorylation sites [46]. Thus, there may be additional cMyBPC phosphorylation changes between the WT and gravin-t/t mice than what we have observed with the three site-specific antibodies that we have used, which may be contributing to the mechanism of increased cardiac function.

Further studies are needed to fully elucidate the mechanism of enhanced cardiac function in gravin-t/t mice. As previously mentioned, gravin contains several other proteins besides PKA in its scaffold that are also involved in the modulation of cardiac function such as PKC. Future experiments will determine the role of these proteins in the mechanism of action. Additionally, using pharmacological reagents to inhibit each of the β-ARs in turn will shed light upon gravin’s effect on their signaling *in vivo*. Furthermore, analysis of ATPase activity, SR load and Ca^2+^ sensitivity will also clarify the mechanism.

## Conclusions

In summary, our results indicate that contractility is enhanced in gravin-t/t mice both with and without β-AR stimulation. The mechanism for this enhanced contractility in gravin-t/t mice may be attributed, in part, to decreased gravin mediated PKA-dependent β_2_-AR phosphorylation. This decreased β_2_-AR phosphorylation would be predicted to decrease receptor desensitization thus accounting for the increased responsiveness to ISO mediated enhanced contractility. However, the mechanism behind the increased basal contractility remains unclear. PKA activity nor PKA phosphorylation of PLB and cTnI were unchanged between WT and gravin-t/t vehicle treated animals indicating that the observed increase in cardiac function during acute β-AR stimulation is not due to increased activity of the SERCA or to cTnI-dependent alterations in myofilament sensitivity. However, phosphorylation of the Ser-273 site in cMyBPC was significantly enhanced in the gravin-t/t mice, which we propose to be involved in either the enhancement of baseline cardiac function or the priming of the myofilaments to increase cardiac function in response to subsequent β-AR stimulation. We also reported that cardiac function remained significantly increased in gravin-t/t mice compared to WT mice in the presence of ISO stimulation. Surprisingly, despite increased contractility in the gravin-t/t mice, PKA activity and phosphorylation of PLB, cTnI and cMyBPC (Ser-282 and Ser-307) was similar between the WT and gravin-t/t mice. However, the PKA substrate Hsp20 showed differential phosphorylation between WT and gravin-t/t mice. Thus, we propose that increased cMyBPC Ser-273 phosphorylation plays a key role in the increased baseline cardiac function seen in gravin-t/t mice and that Hsp20 phosphorylation are involved in the acute ISO stimulated increase in cardiac function in gravin-t/t mice. Taken together, these data indicate that the mice lacking functional gravin protein that does not express the critical region required for binding to β_2_-AR, PKA and PKC increases cardiac contractility by altering PKA phosphorylation of certain substrates as well as the activity of PDE4D. Thus, manipulation of gravin and its scaffolding properties may be a novel method of modulating cardiac function.
